# hEDTA and EDDS as sustainable and harmless alternatives to NTA as trace metal chelators in *Methanothermobacter marburgensis* cultivation

**DOI:** 10.1007/s00792-025-01409-y

**Published:** 2025-11-14

**Authors:** Fabian Schwarzmann, Elisa Hilts, Shubham Gurav, Franziska Steger, Simon K.-M. R. Rittmann, Christian Fink

**Affiliations:** 1 Arkeon GmbH, Tulln a. d. Donau, Austria; 2 ACIB, Austrian Centre of Industrial Biotechnology, Vienna, Austria; 3 Archaea Physiology & Biotechnology Group, Department of Functional and Evolutionary Ecology, Universität Wien, Vienna, Austria

**Keywords:** Microbiology, Biotechnology, Archaea, Methanogens

## Abstract

The thermophilic methanogenic archaea *Methanothermobacter* spp. are applied as archaeal cell factories in industrial processes for methane production. Recently, secretion of commodity chemicals by *M. marburgensis*, such as amino acids, organic acids, or lipids has been observed. Together with the genetic tools available, genetically engineered cell factories for commodity chemical production from CO_2_ can be developed. However, the toxicity of the commonly used trace metal chelator Nitrilotriacetic acid (NTA) for *M. marburgensis* blocks potential regulatory approval for human applications. Therefore, we identified EDDS and hEDTA as suitable non-toxic alternatives as metal chelators for *M. marburgensis*. While EDDS reduces the specific growth rate (µ) of *M. marburgensis* by 40%, hEDTA shows the same µ as with NTA usage. Additionally, hEDTA offers cost wise a sustainable economic alternative to NTA with 23 € m^− 3^ of growth medium compared to 13 € m^− 3^ with NTA. With hEDTA as a harmless alternative to NTA, we are one step closer to industrial sustainable commodity chemical production from CO_2_ with *M. marburgensis*.

## Introduction

Chelating agents are versatile substances that are widely utilized in analytic, scientific, industrial, or medical applications due to their ability to form stable complexes with metal ions. In medicine, chelators play a crucial role in the treatment of heavy metal poisoning by binding toxic metals such as lead, mercury, and arsenic, to facilitate their safe excretion from the human body (Crichton et al. 2016). Furthermore, chelating agents are commonly used for analytical metal detection and quantification in drinking water, soil remediation, and removal of heavy metals from wastewater streams (Byegård et al. 1999; Kociałkowski et al. 1999; Nyamato 2023; Xing et al. 2019). The food industry employs chelators to prevent metal-catalyzed oxidation, thereby enhancing product stability and shelf life (Ogiwara et al. 2016). Additionally, chelators are an integral part of industrial processes such as textile manufacturing and paper production (Goto et al. 2023). In agriculture, chelating agents improve trace metal availability in plants, ensuring efficient uptake of those essential minerals. As a side effect in plant treatment with ethylenediaminetetraacetic acid (EDTA) as chelator, it has been demonstrated that the bioavailability of trace metals is then reduced for soil microorganisms (Kaurin et al. 2020; Lee and Sung 2014).

This reduced bioavailability has also been applied to *Escherichia coli* cultivation, where trace zinc starvation was introduced through addition of EDTA to the minimal medium due to its high binding affinity of zinc to reduce its bioavailability and activate specific biosynthetic pathways for putative biotechnological application (Paterson et al. 2023). Similarly, EDTA has also been shown to inhibit growth of methanogenic archaea in anaerobic digesters (Pham 2017; Vintiloiu et al. 2013). On the other hand, chelating agents can also have beneficial effects on the bioavailability of trace metals (Thanh et al. 2016). Those chelating agents are commonly used in cultivation of microorganisms which are dependent on high abundance of heavy metals, such as acetogenic bacteria or methanogenic archaea (Abdel Azim et al. 2017; Choong et al. 2016). Especially methanogenic archaea have a need for relatively high concentrations of iron, nickel, or cobalt due to their unique set of enzymes that enable growth on molecular hydrogen (H_2_) and carbon dioxide (CO_2_) (Lyu et al. 2018). Commonly, nitrilotriacetic acid (NTA) is used as a chelating agent for pure cultures of methanogenic archaea (Zeikus and Wolfe 1972). The growing interest in industrial applications of methanogenic archaea as cell factories for human-applied commodity chemicals from CO_2_, however, makes the use of NTA for regulatory approval nearly impossible due to its toxic and carcinogenic characteristics (Hartwig 2022). Alternatives to NTA, such as EDTA, ethylenediaminedisuccinic acid (EDDS), hydroxyethylethylenediaminetriacetic acid (hEDTA), or citric acid have been successfully proven to act as harmless chelating agents for fermentation in mixed cultures (Fabbricino et al. 2013; Lanigan et al. 2002; Li et al. 2023; Thanh, Ketheesan, Yan, et al., 2017; Zhang et al. 2021).

For the model microorganism in methanogenic archaea, *Methanothermobacter marburgensis*, which is already applied in large scale industrial processes for methane production in power to gas systems (Götz et al. 2016), an alternative to NTA has not been implemented before to our knowledge. *M. marburgensis* is an aquatic thermophilic methanogenic archaeon isolated from anaerobic sewage sludge in Germany with an optimal growth temperature at 65 °C. Due to its robust cell-wall characteristics and planktonic growth behavior it is highly robust and resilient to shear forces which facilitate bioreactor fermentation (Bernacchi et al. 2014; Seifert et al. 2014). Besides its use as cell factory for methane production, it has been observed that *M. marburgensis* naturally secretes amino acids (Reischl et al. 2025; Taubner et al. 2023). Additionally, with the recently established tools for genetic engineering, it has already been proven that synthetic biosynthesis pathways can be integrated into the genome of *M. marburgensis* to alter the secretion pattern of amino acids (Klein et al. 2025). This marks the first step towards commodity chemical production with *M. marburgensis* and urges the need for a sustainable alternative to NTA as trace metal chelator.

In this study, we aimed to identify sustainable and non-toxic trace metal chelators for the prospective application in industrial processes with *M. marburgensis*. Therefore, we first analyze the stability of trace metal stock solutions and their interactions with the salt medium with other chelators applied. In the second step, we measure the influence of suitable metal chelators and concentrations thereof on growth of *M. marburgensis* and draw conclusions on the commercial feasibility to deviate from NTA to sustainable alternatives.

## Results and discussion

### The non-toxic chelators hEDTA and EDDS allow stable, precipitation-free media for *M. marburgensis*

In a preliminary literature survey, our goal was to identify heavy metal chelators that match the chelating characteristics of nitrilotriacetic acid (NTA) but show enhanced biodegradability without toxic effects on humans. We found five suitable chelating agents for further analysis, which are citric acid, ethylenediaminetetraacetic acid (EDTA), diethylenetriaminepentaacetic acid (DTPA), ethylenediaminedisuccinic acid (EDDS), and hydroxyethylethylenediaminetriacetic acid (hEDTA). The survey already suggested to exclude EDTA and DTPA from the study due to their high metal chelating affinity which normally results in reduced bioavailability of trace metals to microorganisms (Byegård et al. 1999). Nevertheless, we decided to include EDTA as it is one of the best-known, cheapest, and most applied metal chelators in various applications (Cherian et al. 2023). Vice versa, citric acid was estimated as a weak trace metal chelator which was very likely not suitable for chelating high iron and nickel concentrations (Hu et al. 2008). EDDS and hEDTA have both been shown to act as suitable metal chelators in anaerobic digester systems (Li et al. 2023; Pham 2017; Thanh, Ketheesan, Yan, et al., 2017; Zhang et al. 2015). Additionally, their high affinity towards iron, nickel, cobalt, and molybdenum matches the requirements of *M. marburgensis* precisely. While hEDTA, similar to EDTA shows high stability in biological systems and is therefore not rendered biodegradable (Hinck et al. 1997; Sýkora et al. 2001), EDDS was proven to efficiently biodegrade (Schowanek et al. 1997). Furthermore, photoinactivation of EDDS has been successfully applied (Metsärinne et al. 2001). EDTA is in doubt for toxic effects at high concentrations. Here, hEDTA and especially EDDS are beneficial since no toxic effects have been observed for use in human-applied products (Cherian et al. 2023; Fabbricino et al. 2013; Lanigan et al. 2002).

It has been shown that precipitation-free trace metal stock solutions for closed batch cultivation of methanogens can be generated in highly acidic milieu (pH < 1) (Fink et al. 2021). The application of those acidic trace metal stock solutions to large-scale bioprocesses imposes negative effects. (I) The required continuous feed of trace metals or addition of repetitive shots influences the local and global pH of the bioreactor system. (II) The concentration required for high cell density cultivation requires trace metal concentrations that by far exceed the concentrations in closed batch systems which exceed the solubility limit in the fermentation broth (Simpson et al. 2015). (III) Slightest molecular oxygen contaminations in the anoxic bioreactor system with sodium sulfide as reducing agent cause precipitation of nickel and iron as nickel/iron sulfides (Li et al. 2024). Based on those risks, the addition of chelators to large-scale bioreactor systems for trace metal complexing is obligate.

**Fig. 1:**
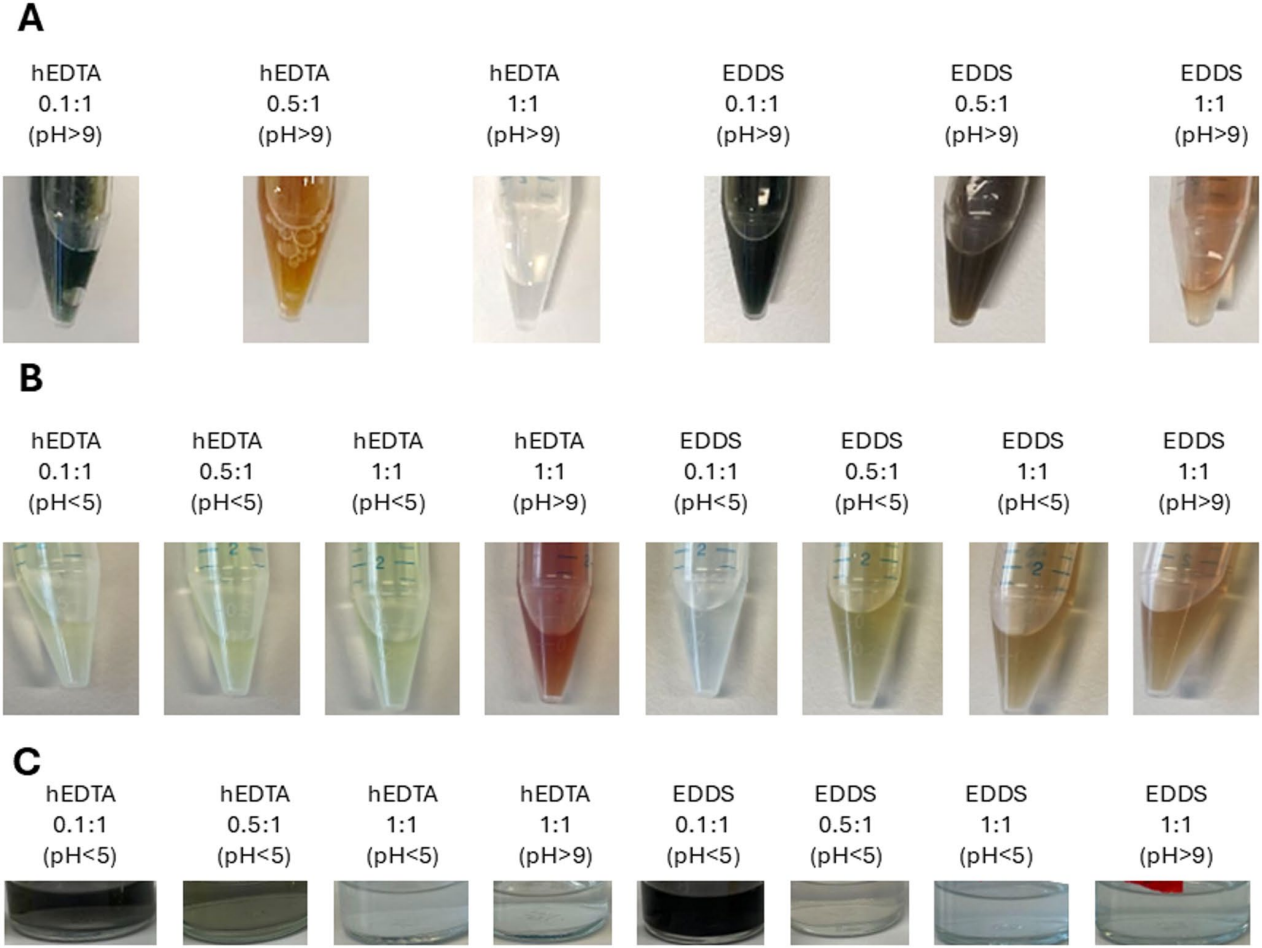
Impact of pH and autoclavation on trace metal stock solutions with differing concentrations of EDDS and hEDTA and their overall effect on reduced basal medium for cultivation of *M. marburgensis.* **A**) 0.1:1, 0.5:1, and 1:1 molar ratio of EDDS and hEDTA with the standard trace element stock solution composition at pH > 9. **B**) Stable trace metal stock solutions at different pH and molar ratios after autoclavation. **C**) Reduced basal medium for cultivation of *M. marburgensis* after addition of different trace metal stock solutions

Before we initiated biological growth experiments with *M. marburgensis*, we needed to ensure that the trace metals in stock solutions were non-precipitating in the presence of the chelator. Additionally, we studied the impact of basic and acidic milieu (pH > 9, pH < 5) and autoclavation on the stability of the trace metal stock solutions. We always started from a 1:1 molar ratio of the new chelating agent to trace elements with a concentration of 0.11 mol L^− 1^ in the stock solution as it is the common practice for NTA usage (Abdel Azim et al. 2017; Zeikus and Wolfe 1972). While the addition of EDTA, hEDTA, and EDDS in a 1:1 molar ratio independent of the pH resulted in binding of all trace metals, citric acid had not enough binding capacity and resulted in trace metal precipitation in the stock solution (data not shown). To avoid acidification of the media by adding trace metal stock solution, we tested the impact of pH > 9 on the stock solution of EDTA, hEDTA, and EDDS. Here we found significant differences. At a pH < 5 all chelating agents kept the metals in complete solution at a molar ratio of 1:1. For the hEDTA and EDDS solutions, the ratio could even go as low as 0.1:1. As the pH increased a molar ratio of 1:1 proved necessary to keep the metals from precipitating (Fig. 1A). We continued with non-pH controlled 0.1:1, 0.5:1, and 1:1 molar ratio and 1:1 ratio of pH > 9 EDDS and hEDTA stock solutions, proved that they were stable after autoclavation, and tested their impact on the sodium-sulfide reduced medium of *M. marburgensis* (Fig. 1B and C). 0.1:1 molar ratio of EDDS and hEDTA, both turned black as iron(II)-sulfide precipitation was formed as a reaction of sulfide and insufficient iron-chelating capacity (Pessu et al. 2021). In 0.5:1 molar ratio hEDTA and EDDS showed still precipitation but less compared to 0.1:1 which rendered 0.5:1 EDDS as usable for cultivation. In 1:1 molar ratio independent of the pH of the stock solution no precipitate was formed (Fig. 1C). A 1:1 molar ratio of EDTA was also tested and resulted in precipitation-free medium (Schwarzmann et al. 2025).

### A 1:1 molar ratio of hEDTA results in identical growth rates of *M. marburgensis* compared to NTA

After we identified the 1:1 molar ratio of hEDTA and trace metals (0.11 mol L^− 1^ in 1000x stock solution) as a suitable concentration for the use in reduced basal medium for *M. marburgensis* cultivation, we continued with qualitative growth experiments with *M. marburgensis*. Therefore, we inoculated *M. marburgensis* to an optical density of ~ 0.05 and incubated at 62 °C until growth occurred. After 3–4 days of adaptation, we observed growth with 1:1 molar ratio of EDDS and hEDTA. However, with EDTA no growth has been observed even after 14 days of incubation. With that, EDTA was excluded as a chelating agent for further growth experiments.

**Fig. 2:**
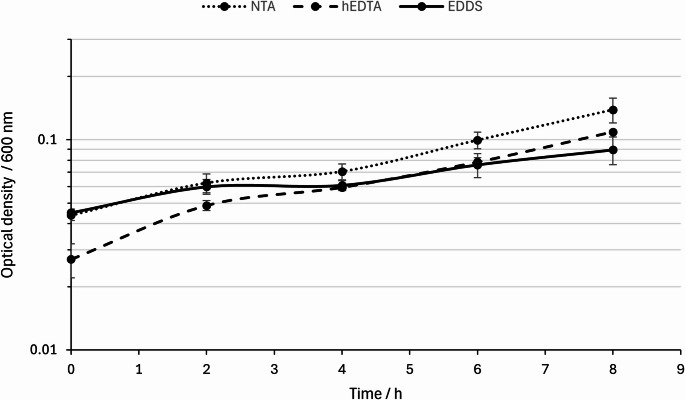
Semi-logarithmic growth curves of *M. marburgensis* over a time frame of 8 h in exponential growth phase with EDDS (solid line), hEDTA (dashed line), and NTA (dotted line) as trace metal chelating agents with a molar ratio of 1:1 compared to the standard NTA concentration in *M. marburgensis* medium. Error bars indicate the standard deviation of biological triplicates (*n* = 3)

In the next step, we compared 1:1 molar ratio of EDDS and hEDTA to NTA containing media and their impact on the growth behavior of *M. marburgensis*. We used the chelator-adapted cultures from the qualitative growth experiment, prepared an additional overnight culture with the respective chelator, and used that preculture for a phenotypic characterization (*n* = 3). We demonstrated stable growth of *M. marburgensis* with 1:1 molar ratio of EDDS and hEDTA. NTA and hEDTA differed in start optical densities, but when the specific growth rates (µ) were calculated, we found no difference between NTA and hEDTA with µ = 0.2 h^− 1^, and µ = 0.2 h^− 1^, respectively (Fig. 2). The growth behavior with EDDS applied, however, was notably reduced compared to NTA and hEDTA with an average growth rate of µ = 0.12 h^− 1^ (Fig. 2).

### Reduction of chelating agent concentrations lead to reduced growth rates of *M. marburgensis*

We already had indication for feasibility of lower molar ratios of EDDS and hEDTA on the solubility of the trace metals when we tested 0.5:1 (Fig. 1C). Hence, we aimed to reduce the molar ratio to a minimum where enough trace metals are still bound and no negative impact on precipitation in the medium was observed already with an economic perspective in mind. For EDDS, the minimal molar ratio was 0.65:1 and for hEDTA 0.75:1, respectively. We performed a subsequent phenotypic characterization in duplicate (*n* = 2) of 0.65:1, 0.85:1, and 1:1 molar ratio of EDDS and 0.75:1, 0.85:1, and 1:1 molar ratio of hEDTA to see the impact on µ of *M. marburgensis*. We measured the maximal specific growth rate (µ_max_) between two measurement points (2 h) and the average growth rate amongst the 8 h of incubation at 62 °C. In the experiments with both EDDS and hEDTA, the global specific growth rate (µ_global_) was reduced by roughly 50% compared to the 1:1 ratio at lower ratios (Table 1). However, µ_max_ of hEDTA was not as affected indicating for an adaptative mechanism or limiting effect (Table 1). In previous studies a positive effect of higher molar rations of EDDS to iron on methane production in anaerobic digesters has been proven which aligns with the results of this study (Thanh, Ketheesan, Stuckey, et al., 2017a; Zhang et al. 2015). Additionally, it has been shown that NTA leads to higher methane production as a proxy for methanogenic archaea activity compared to EDDS (Zhang et al. 2021). It can be deducted that a lower molar ratio of EDDS and hEDTA to trace metals leads to reduced bioavailability of the respective trace metals. While it is known that the high trace metal binding affinity of EDTA reduces the methane production in anaerobic digesters, the impact of hEDTA usage is rather novel. Apparently, the lower binding affinity to metals of hEDTA due to its pentadentate complexation leads to higher bioavailability compared to the hexadentate ligand EDTA (Kociałkowski et al. 1999).

**Table 1:** Average and maximum growth rate of *M. marburgensis* with different molar ratios of EDDS and hEDTA as trace metal chelating agents

Metal chelator/molar ratio	µ_global_/h^− 1^	µ_max_/h^− 1^
NTA	0.20	0.24
1:1 hEDTA	0.20	0.21
1:0.9 hEDTA	0.09	0.16
1:0.75 hEDTA	0.09	0.19
1:1 EDDS	0.12	0.12
1:0.75 EDDS	0.07	0.10
1:0.65 EDDS	0.06	0.08

### hEDTA offers a cost-effective sustainable alternative to NTA

In the study regarding media stability and growth of *M. marburgensis* on the NTA alternatives EDDS and hEDTA, we found a molar ratio of 1:1 compared to the standard NTA molarity of the *M. marburgensis* medium as highly suitable. However, even with lower molar ratios of up to 0.75:1 or 0.65:1 in hEDTA and EDDS, respectively, we identified stable media conditions and reduced but continuous growth of *M. marburgensis* (Table 1). Another important aspect besides the technical feasibility of a trace metal chelator change towards a more sustainable and non-toxic alternative is the economy of the process. This is especially important when establishing industrial processes, where reactor sizes are often more than 100 m^3^. In comparison of the price per kg of the respective chelating agent, NTA is the cheapest substance with 139 € kg^− 1^, followed by hEDTA with 195 € kg^− 1^, and EDDS with 515 € kg^− 1^ of a 35% EDDS containing liquid solution (Table 2). When we applied the different molar ratios compared to NTA and calculated the price for each chelator per m^3^ of *M. marburgensis* media, we ended up with 13 € L^− 1^ for NTA, 28 € L^− 1^ and 21 € L^− 1^ for 1:1 and 0.75:1 molar ratios of hEDTA, and 242 € L^− 1^ and 121 € L^− 1^ for 1:1 and 0.5:1 molar ratios when EDDS is used (Table 2). While 0.75:1 of hEDTA with 21 € L^− 1^ is in a relatively close price range to NTA considering its beneficial effects non-toxicity especially for the downstream processing, EDDS shows a considerable price difference to NTA even with the lower molar ratio of 0.5:1 but has an advantage in biodegradability compared to NTA and hEDTA (Byegård et al. 1999; Schowanek et al. 1997; Sýkora et al. 2001).

**Table 2:** Price calculations per kg and in 1 m^3^ of *M. marburgensis* for NTA and the more sustainable alternatives hEDTA and EDDS with different molar ratios in the medium

Chelator [molar ratio to NTA]	NTA	hEDTA [1:1]	hEDTA [0.75:1]	EDDS [1:1]	EDDS [0.5:1]
Price [€] per kg	139^1^	195^2^	195^2^	515^3^	515^3^
Price [€] per 1 m^3^ medium	13	28	21	242	121

^1^ Purchased from Carl Roth GmbH & Co KG (Karlsruhe, Germany)

^2^ Purchased from Sigma-Aldrich Co. LLC (St. Louis, MO, USA)

^3^ Purchased as 35% trisodium salt solution from Sigma-Aldrich Co. LLC (St. Louis, MO, USA)

In conclusion, both hEDTA and EDDS both offer suitable alternatives to NTA for cultivation of *M. marburgensis*. hEDTA results in similar growth rates of *M. marburgensis* compared to NTA usage and has a relatively close price range per m^3^ of *M. marburgensis* media. EDDS usage results in lower growth rates, has higher costs of application, but offers the great advantage of biodegradability. Which chelating agent is ultimately used for a specific fermentation process depends on the intended application. Both hEDTA and EDDS can be used as safe alternatives to NTA which makes them especially suitable for human-product related applications.

## Materials and methods

### Strain, media, and cultivation conditions

We defined *M. marburgensis* Δ*hpt* as strain of choice for this study and referenced it as wild type throughout the manuscript (Klein et al. 2025). We prepared *M. marburgensis* media with the following salt content: NH_4_Cl, 2.1 g L^− 1^; K_2_HPO_4_, 6.8 g L^− 1^; NaHCO_3_, 3.6 g L^− 1^, 6 mL trace element (TE) solution (1000x), and 1 mL of resazurin sodium salt solution (0.025%) (Abdel Azim et al. 2017; Klein et al. 2025). Afterwards, we set the pH to 7.2 with 10 mol L^− 1^ sodium hydroxide. To remove soluble molecular oxygen from the medium but keep the carbonate buffer in place, we gassed with molecular nitrogen/CO_2_ (20 Vol.-% CO_2_ in molecular nitrogen) for 40 min and transferred the anoxic medium into the anaerobic chamber (Coy Laboratory Products, Grass Lake (MI), USA). For further reduction of oxidized compounds, the medium was reduced inside the anaerobic chamber by addition of 2 mL L^− 1^ of a 0.5 mol L^− 1^ Na_2_S·9H_2_O-solution. When the medium turned colorless due to reduction of the resazurin redox indicator, we anoxically filled 25 mL per 120 mL serum bottle of *M. marburgensis* medium. After sealing the serum bottles with butyl stoppers and aluminum crimps, we exchanged the headspace to H_2_/CO_2_ (4:1) (20 Vol.-% CO_2_ in H_2_) with 3 cycles of −0.8 bar and 1 bar overpressure with a final pressure of 1 bar H_2_/CO_2_ (4:1) gas mixture. The purity of all gases was 5.0 (99.999%). We autoclaved ready-made media bottles at 121 °C for 20 min and 1.2 bar above atmospheric pressure in a vertical autoclave (HMC Europe GmbH, Tüssling, Germany).

### Generation of trace metal stock solutions

The trace element stock solutions (TE) consisted of MgCl_2_·6H_2_O 2 g L^− 1^, FeCl_2_·4H_2_O 10 g L^− 1^, CoCl_2_·6H_2_O 0.01 g L^− 1^, NiCl_2_·6H_2_O 2.4 g L^− 1^, NaMoO_4_·2H_2_O, 0.01 g L^− 1^ and is defined as 6xNSTE (Haslinger, Reischl et al., Communications Biology, in revision). As chelating agent for the TE solutions, we traditionally used 90 g L^− 1^ of Nitrilotriacetic acid (NTA) at pH of 6.4 which resembles a 1:1 molar ratio of trace metals to NTA. We calculated the same 1:1 molar ratio of trace metals for alternative chelating agents (Eq. 1). This resulted in 40 g L^− 1^ of EDTA, 10 g L^− 1^ of citric acid, 29.88 g L^− 1^ of hEDTA, and 109.9 mL L^− 1^ of a 35% solution of EDDS. We tested pH adjusted conditions of the TE stock solution by titrating the pH to above 9 with NaOH. Additionally, we tested conditions without pH adjustment that resulted in pH < 5. The TE stock solutions were sterilized by autoclavation at 121 °C for 20 min and 1.2 bar above atmospheric.

Calculation chelator concentration based on molar ratio compared to trace metals (Eq. 1)

1 $$ \:\left( \begin{gathered} m_{{Mg}} *M_{{Mg}} + m_{{Fe}} \hfill \\ *M_{{Fe}} + m_{{Co}} *M_{{Co}} \hfill \\ + m*M + m_{{Mo}} *M_{{Mo}} \hfill \\ \end{gathered} \right)*1000*M_{{Chelator}} = C_{{Chelator}} $$

### Phenotypic characterization

We did closed batch phenotypic characterizations with 25 mL of *M. marburgensis* media in 120 mL serum bottles with the trace element composition given above and 2 bar overpressure of H_2_/CO_2_ (20 Vol.-% CO_2_ in H_2_) as discussed in Klein et al. (2025). We generated overnight cultures of *M. marburgensis* adapted to the respective trace metal chelator and molar ratio compared to trace metals. From those, we inoculated day cultures in biological triplicates to an OD_600_ = 0.05. We incubated the triplicates at 62 °C, shaking at 170 rpm (Eppendorf New Brunswick Innova, Hamburg, Germany). We measured the OD_600_ every 2 h for at least 8 h of incubation with a spectral photometer (Hach Lange GmbH, Berlin, Germany).

Based on the results from optical density measurements at 600 nm, we calculated the specific growth rate (µ) / h^− 1^ of *M. marburgensis* per h (Eq. 2). From the 5 time points where the optical density was measured, after 0, 2, 4, 6, and 8 h, we calculated global specific growth rate (µ_max_) / h^− 1^ between time point 0 h and 8 h. The maximum specific growth rate (µ_max_) / h^− 1^ was defined as the highest growth rate between two measurement time points.

Calculation of µ / h^−1^ (Eq. 2)

2 $$\:\frac{\left(\frac{{OD}_{end}-{OD}_{start}}{{OD}_{start}}\right)*100}{{Hours}_{Incubation}}$$

## Data Availability

All data generated or analyzed during this study are deposited at the University of Vienna PHAIDRA system accessible via https://doi.org/10.25365/phaidra.696.
